# Obstetrical Soft Tissue Trauma during Spontaneous Vaginal Birth in the Romanian Adolescent Population—Multicentric Comparative Study with Adult Population

**DOI:** 10.3390/ijerph182111491

**Published:** 2021-10-31

**Authors:** Alexandra Matei, Elena Poenaru, Mihai Cornel Traian Dimitriu, Cristina Zaharia, Crîngu Antoniu Ionescu, Dan Navolan, Cristian George Furău

**Affiliations:** 1 Department of Obstetrics and Gynecology, “Carol Davila” University of Medicine and Pharmacy Doctoral School, 020021 Bucharest, Romania; antoniuginec@yahoo.com; 2 Department of Biostatistics, “Carol Davila” University of Medicine and Pharmacy, 050474 Bucharest, Romania; 3 Department of Obstetrics and Gynecology, “Sf. Pantelimon” Emergency Clinical Hospital, 021659 Bucharest, Romania; mihai.dimitriu@umfcd.ro (M.C.T.D.); zh_cristina@yahoo.com (C.T.); 4 Department of Obstetrics and Gynecology, Victor Babes University of Medicine and Pharmacy, 300041 Timisoara, Romania; navolan@yahoo.com; 5 Department of Obstetrics and Gynecology, Emergency County Hospital, 310037 Arad, Romania; cristianfurau@gmail.com

**Keywords:** episiotomy, vaginal birth, perineal trauma, soft tissue injury, birth laceration, adolescent birth

## Abstract

Romania is a country with high rates of adolescent births, associating scarce comprehensive obstetrical management with this specific population. This research aims to assess soft tissue trauma after vaginal birth in teenage mothers compared to their adult counterparts. A retrospective case-control study was conducted for one year in two hospitals. All vaginal deliveries were considered; the age cut-off value was considered at 20 years old for case and control groups. Lacerations were divided into three subgroups, considering the involved anatomical region; group I: labial and periurethral lacerations, group II: vaginal and perineal lacerations, and group III: cervical lacerations. There were 1498 women included in the study: 298 young mothers and 1200 adults. Teenagers were more likely to have an episiotomy during vaginal delivery compared to adult women: 56% versus 26.7% (*p* = 0.00, Pearson Chi-square) and a 1.89 times increased risk for developing additional group II lacerations: *p* = 0.01, Pearson Chi-square test with Bonferroni correction: OR = 1.89, 95% CI: 1.18–3.02. Group II lacerations were the most frequent type of birth trauma in both study groups. Fetal weight ≥4000 g was associated with a two times higher risk for vaginal and perineal lacerations when age criterion was not considered (OR = 1.98, 95% CI: 1.13–3.47, *p* = 0.01). The incidence of group I and II lacerations increased with age: from 0% and 9.1% between 10 and 14 years old to 6% and 26.2% between 18 and 19 years old. All groups of lacerations were more often identified in the case group, compared to the adult group. Fetal macrosomia and spontaneously ruptured membranes at admission could not be documented as risk factors for obstetrical injury in young mothers. Episiotomy performed in teenagers was not a protective procedure for group II lacerations.

## 1. Introduction

The age of European women who bear children varies widely. Although related to different etiologies, maternal morbidity and mortality rates are highest among the youngest and oldest women [1]. Statistical reports show that 9.7% of Romanian births in 2015 were attributed to women younger than 20 years of age while at the same time, having children later in life is a general trend across the entire continent [1].

In Romania, vaginal births account for 55.9% of all deliveries although the country ranks second after Cyprus among European Union countries concerning rates of caesarean sections [2]. Evidence shows that countries reporting high rates of caesarean deliveries have very low rates of instrumental vaginal delivery; this is also the case in Romania, where a rate of 0.5% of instrumental deliveries is reported [3]. Pregnancy represents a physiologic process and interventions during the labor and expulsive phase can negatively influence the birth experience. Giving birth is a major transition in the lives of women; teenage mothers perceive vaginal birth as a stressful experience, mainly because of pain [4]. Furthermore, the performance of episiotomy was found to be related to maternal emotional disturbances [5].

Although over the past years the mean age of Romanian women at the birth of the first child has been constantly increasing, 11.2% of first-born babies in 2019 belonged to mothers of 10 to 19 years of age [6,7]. Teenage mothers represent a vulnerable group for the development of high-grade perineal tears during spontaneous vaginal birth [8].

Almost 85% of vaginal births are associated with some form of trauma to the genital tract irrespective of its nature: spontaneous or iatrogenic via episiotomy or instrumental delivery, while approximately 6% of women will experience a severe perineal laceration [9,10,11]. Routine use of episiotomy is no longer promoted; the need for fastened delivery of a compromised fetus, operative vaginal delivery, soft tissue dystocia, increased risk for severe perineal tears, and history of female genital mutilation are main indications of justified episiotomy [10,12].

Perinatal maternal morbidity is related directly to the degree of perineal injury sustained [9]. Pelvic floor trauma during birth can be responsible for localized pain, sexual dysfunctions, urinary and anal incontinence, involving further psychological and social disruptions that can disturb the woman’s ability to care for her baby [9,13]. Previous studies have identified primiparity, fetal weight higher than 4000 g, operative delivery, the prolonged second stage of labor, and shoulder dystocia as the most often cited risk factors for severe lacerations [14]. Specifically, in the adolescent population, risk factors for perineal tears of primiparity, fetal position, gestational diabetes, and duration of the second stage of labor have already been documented [8]. The long-term complications associate medico-legal repercussions and increased health care costs, especially since studies on young women with pelvic floor symptoms proved that they may be at risk for increased severity of these conditions later in life [8,15].

The problem of perineal trauma during labor and spontaneous birth has already been addressed in literature based on age-related anatomical characteristics, racial or ethnic disparities, and obstetrical complications. However, Romania remains a country with high rates of adolescent births, where comprehensive obstetrical management in this specific population of women is scarce.

The primary outcome of this study consists in investigating perineal trauma during spontaneous vaginal birth in teenage women who gave birth in two public hospitals from Romania, by comparing them to adult women; additionally, we aimed to identify age-specific risk factors for obstetrical perineal tears by analyzing adolescent spontaneous birth injuries compared to those identified in adult women.

## 2. Materials and Methods

This is an observational retrospective comparative study conducted over 12 months, March 2020 to March 2021, in two medical institutions from Romania: “Sf. Pantelimon” Emergency Hospital from Bucharest, a tertiary level unit, and Arad County Emergency Clinical Hospital, a regional hospital, accounting for approximately 1600 and 2600 births per year, respectively.

All patients who delivered in the Department of Obstetrics during the study period were considered for inclusion in the study. In Romania, birth is considered starting 24 weeks of gestation and consequently all study participants were at ≥24 weeks of gestation at the time of vaginal birth. Subdivision of patients with preterm versus term pregnancies was not performed since this could lead to potentially biased results with respect to the practice of episiotomy. Young women aged under 16 years old were automatically monitored during hospital admission by investigators from the social services and child protection services division.

Exclusion criteria referred to vaginal births without medical assistance and deliveries through caesarean section. Medical records and birth registries were primary sources of demographic data: age; provenance environment: urban or rural settings; and medical and obstetrical history, with a focus on diabetes: pre-existing or gestational diabetes. For the included patients, the following maternal variables were documented: parity; gravidity; type of pregnancy: single or multiple; fetal presentation: cephalic or breech; membrane status at admission time: intact or ruptured; whether episiotomy was performed; presence of perineal tears and lacerations; instrumental delivery; and length of hospital stay. Fetal numeric variables were also collected: Apgar score at 5 min and fetal weight.

Only patients already diagnosed with diabetes at the time of admission for birth assistance were considered as having this affection. Rural settlements were the smallest administrative-territorial units, characterized by a reduced density of population, where agricultural occupations are performed. We considered “spontaneous ruptured membranes” diagnosed at admission as a potential risk factor for soft tissue tearing in the context of reduced prenatal follow-up and predisposition to inflammation and/or infection.

The age cut-off value was considered at 20 years old, following the World Health Organization (WHO) recommendations. The study was based on two age-differentiated groups of women: patients under 20 years old were adolescents and formed the “case group”. Women aged 20 years old and above were considered adults and formed the “reference/control group”. We also analyzed the adolescent group in-depth, comparing their laceration outcomes in three specific age subgroups: 10–14 years old corresponding to early adolescence, 15–17 years old corresponding to middle adolescence, and 18–19 years old corresponding to late adolescence, as the WHO stated [16].

All the deliveries in the two centers were performed in the lithotomy position. Patients who had episiotomy also had corresponding surgical repair; only mediolateral incisions were performed. Women who had an episiotomy performed were classified as “episiotomy”; if concomitant lacerations were identified, they were also considered.

For research purposes, obstetrical lacerations were divided into three subgroups, considering the involved anatomical region: group I: injuries comprising labial and periurethral region; group II: lesions involving vaginal mucosa and the perineum; and group III: injuries of the cervix. Perineal tears involving the anal sphincter (third- and fourth-degree tears) were assessed independently.

Statistical analysis was performed using SPSS Software (IBM Corp., Armonk, NY, USA) and Microsoft Excel data analysis tool (Microsoft 365 Personal). Variables were tested for normality using both Shapiro–Wilk and Kolmogorov–Smirnov test depending on the sample size. Quantitative variables were presented using means and corresponding standard deviations; categorical variables were described using frequencies and percentages and were compared using Fisher’s exact test and Chi-square test. The Odds ratio (OR) with corresponding 95% confidence interval (CI) estimated the degree of exposure of case and control groups to different categorical variables. Predictive models of risk and protective factors were assessed using univariate and multivariate logistic regression. A *p*-value below 5% was considered statistically significant.

This research was conducted according to the guidelines of the Declaration of Helsinki and approved by the Institutional Ethics Committee of “Sf. Pantelimon” Emergency Hospital (2134/21.01.2020) and of Arad County Emergency Clinical Hospital (1815/28.09.2020). Data were collected anonymously and exclusively for research purposes.

## 3. Results

There was a total of 1498 women included in the study: 298 young mothers and 1200 adult patients from both hospitals. The fractions of included deliveries from each facility department were 40.8% from the tertiary unit and 59.1% from the regional unit. The proportions of adolescent mothers included in the report were similar for both health centers: 21.0% from “Sf. Pantelimon” Emergency Hospital and 19.0% from Arad Regional Hospital.

The mean age in the two study groups was significantly different: 17.3 ± 1.5 years old in the case group versus 28.4 ± 5.8 years old in the control group (*p* < 0.00, independent sample test). Demographic and obstetrical characteristics of patients in the two study groups are presented in Table 1.

The case-control data analysis showed that the incidence of multiple pregnancies, (0.0% versus 0.1%), fetal breech presentation (0.3% versus 0.7%), ruptured membranes at admission (17.1% versus 18.9%), and history of diabetes (0.3% versus 0.4%) were similar in the adolescent mothers compared to the adult reference population. Teenage mothers gave birth to fetuses with lower birth weight compared to those born from adult mothers: 2976.5 ± 471.7 g compared to 3149.5 ± 518.6 g, *p* = 0.00. Neonates from young mothers had Apgar score ≤7 more often compared to those from adult mothers, 7.4% versus 4.8%, but results were not statistically significant: *p* > 0.05, Pearson Chi-square. On average, adolescents had longer admissions compared to adult women: 4.4 ± 3.4 days versus 3.8 ± 2.8 days (*p* = 0.001, Independent Samples Test).

Comparing teenagers in middle adolescence with those in late adolescence, we noted that there were significant differences between the proportions of primiparas: 79.7% versus 55.7% and two-paras: 15.9% versus 33.6% (*p* = 0.00069, likelihood ratio); also, there were significantly more primigravidas in early adolescence versus late adolescence groups, 100% versus 49%, and between middle adolescence mothers and late adolescence mothers, 71% versus 49% (*p* = 0.001, likelihood ratio).

As seen in Table 2, teenage mothers were more likely to have an episiotomy during spontaneous birth compared to adult women: 56.0% versus 26.7% (*p* = 0.00, Pearson Chi-square). Adolescents were more likely primiparas, 68.1%, compared to adult mothers, 23.3% (*p* = 0.00). However, there was no significant difference between the proportion of adolescent versus adult primiparas who had an episiotomy: 68.4% versus 68.8% (*p* = 0.93, Pearson Chi-square). A significantly higher proportion of adult mothers had intact soft tissues after spontaneous birth compared to adolescent mothers: 49.8% compared to 25.2% (*p* = 0.00, Pearson Chi-square). For women younger than 20 years old who had an episiotomy, we found a 1.89 times increased risk for developing additional group II lacerations: *p* = 0.01, Pearson Chi-square test with Bonferroni correction: OR = 1.89, 95% CI: 1.18–3.02.

Group II lacerations were the most frequent type of birth trauma in both study groups (Table 2). There were no suggestive differences between the incidence of soft tissue traumas between young mothers in middle versus late adolescence (*p* = 0.06, Pearson Chi-square). All three groups of lacerations were more often identified in the teenage group compared to the adult group, but this variation had no statistical significance. A proportion of 40.8% of young primiparas had lacerations compared to 31.6% of their adult counterparts, but this variation was not meaningful between the two groups (*p* = 0.56, Pearson Chi-square).

Among all study participants for each group of lacerations, we performed a univariate logistic regression to identify potential risk factors, followed by a multivariate regression analysis of the specific variables. Primiparity was not a predictive factor for group I labial and periurethral lacerations, *p* = 0.22, or for group II vaginal and perineal lacerations, *p* = 0.09.

The multivariate regression analysis identified the following variables as protective factors for group I lacerations: the practice of episiotomy (*p* = 0.00, OR: 0.15, 95% CI: 0.05–0.44) and the adult maternal age (*p* = 0.02, OR: 0.46, 95% CI: 0.23–0.92).

In the study group, primiparas were 1.53 times more likely to develop cervical lacerations compared to multiparas (OR = 1.53, 95% CI: 1.02–2.28, *p* = 0.03) but the results could not be confirmed when the analysis was performed on age-depending groups (Table 3). Fetal weight ≥4000 g was also assessed as a possible risk factor for soft tissue trauma during vaginal birth and we identified a two times higher risk of group II lacerations when age criterion was not considered (OR = 1.98, 95% CI: 1.13–3.47, *p* = 0.01). Similar results were obtained in the adult reference group: OR = 1.98, 95% CI: 1.08–3.62, *p* = 0.04. Fetal macrosomia did not prove to be a risk factor for obstetrical injury in the adolescent population overall or in any of the adolescent subgroups which were evaluated. There was no significant difference between patients with intact versus ruptured membranes regarding all three groups of lacerations.

There were no instrumental deliveries registered for the women included in the project. All patients previously diagnosed with diabetes had group II lacerations, but this medical condition could not be significantly correlated with vaginal or perineal injuries (*p* > 0.05).

No women included in the study were diagnosed with a fourth-degree perineal tear; third-degree tears were identified in 0.3% of parturient women and all of them belonged to the adult group.

## 4. Discussion

Perineal tears are common among women who have spontaneous birth and their association with factors like maternal gynecological and obstetrical characteristics, interventions during labor and delivery, and fetus-related aspects have been extensively analyzed in literature [17,18,19]. In this report, facing a persistent national concern regarding adolescent birth rate, researchers have sought to identify the impact of maternal age on the incidence of birth-related soft tissue trauma, by focusing on teenage mothers.

Women experience more than one risk factor during delivery. Studies regarding the impact of maternal age on the risk of developing obstetrical perineal trauma are conflicting; description of soft tissue outcome after vaginal birth also implies extensive consideration of maternal race, ethnicity, parity, and labor interventions [10,13,20,21].

Although there is inconsistent evidence that low school achievement is a risk factor for perineal trauma [18,22], the fact that 60% of women included in this report came from rural settings where there is limited access to education resources and healthcare services suggests that social, educational, and environmental factors play relevant roles in the obstetrical outcome, as discussed in previous studies [23].

In our study, we have shown that the incidence of lacerations during adolescence increases with age; this is contrary to some reports suggesting that the risk of birth trauma is higher in younger adolescents compared to older teens [24]. Furthermore, all laceration groups were more often identified among young mothers compared to their older counterparts. This is contrary to the theory that advanced maternal age is associated with adverse perineal outcomes [10,20,21] but is consistent with the increased length of hospital stay that we have identified in young women.

The healthcare framework appraised in this study, which we consider to be evocative for the Romanian obstetrical practice, presents particular but relevant aspects to be considered before interpreting our results: decreased adolescent maternal age at birth, with lower educational attainment and scanty instrumental deliveries. Risk stratification of these patients, when assisted for spontaneous birth, is a powerful indicator of their obstetrical outcome. The selective episiotomy policy in Romania is not supported by national protocols and a firm practical basis.

There is a larger body of evidence to support forceps and vacuum delivery, use of midline episiotomy, and increased fetal birth weight as the strongest risk factors for anal sphincter injuries [25,26,27]; this validates our findings on the reduced incidence of severe perineal lacerations in the absence of operative deliveries and practice of only mediolateral episiotomy. Fetal macrosomia was featured in our results as a risk factor for group II lacerations among adult mothers, but this finding could not be verified for teenage mothers as well; we interpreted these results as concurrent with the fact that neonates delivered by young women had significantly lower birth weight compared to those delivered by adult mothers. However, in this situation, gestational age must be considered as a potential confounding variable; a recent populational study denoted that prematurity reduced the risk of episiotomy both in teenage mothers and in patients 30 to 39 years of age [28] but in our country, particularly in preterm deliveries, episiotomy remains a very popular practice [29].

Our results showed that adverse neonatal outcome marked by Apgar score 1 to 7 has been associated more often with young mothers than with adults: 7.4% compared to 4.8%. These findings corroborated those reported by Egbe et al., who considered that a possible explanation to this aspect could reside in the small birth canal, rigid perineum, and inappropriate psychological preparation for labor and delivery [30]. In other reports, a strong association between episiotomy practice and Apgar score [29] could be established. There is great variability in the size, symmetry, and morphology of the vulvar structures in the adolescent population [31] but shortened perineal length (<25 mm) has been reportedly involved in perineal tearing [12,32]. Additionally, birth in lithotomy positioning—the exclusive position for vaginal birth used in our research—has already been identified as an intrapartum risk factor for perineal tears [12].

This research focused on young mothers, showing them to have a particular pattern of lacerations; although during adolescence the incidence of lacerations increased with age in observed groups, most often, these parturient women had group II lacerations, involving vaginal and perineal structures. When adjusted for parity, we found that young primiparas more often had group III lacerations involving the cervix. These results are still inconsistent with other studies, reporting group I lacerations to be more frequent among primiparous teenage mothers [33].

Analysis of episiotomy use in the cohort revealed a significant difference between young and adult mothers; compared to previous Romanian reports [29], the incidence of this surgical procedure among mothers ≥ 20 years old has decreased to below 30%, which is in agreement with WHO recommendations [17]. Contrastingly, 56% of the adolescent population in our research had an episiotomy during birth but at the same time, the proportion of primiparas in this group was significantly higher compared to the adult group: 68.1% versus 23.3%. The increased episiotomy rate is concerning since short- and long-term complications and consequences are worse among women who underwent an episiotomy compared to those who developed tears [34], even more concerning if we consider that the practice of episiotomy in the case group did not provide protection for vaginal and perineal tearing.

In recent years, episiotomy rates underwent a significant reduction worldwide along with a slight increase in perineal tears [5,28]. In a previous European study, Blondel et al. identified a significant negative correlation between episiotomies and third- and fourth-degree perineal tears [35]; our results on the association between these variables show the persistence of an inversely proportional relationship, augmented in the young mothers group, where no severe lacerations were identified but episiotomy reached rates as high as 56%. Conversely, researchers are reporting an increased susceptibility of young mothers from developing countries to third- and fourth-degree tears, but evidence of episiotomy use was not included [36].

A proportion of 0.4% of patients included in the study had a history of diabetes but no valid correlations could be established with perineal trauma. However, gestational diabetes requiring insulin use for control continues to pose a 6.5 times higher risk for a severe perineal laceration in the adolescent population, as outlined by Patterson et al. [11]. Regarding the presence spontaneous ruptured membranes during admission at the labor room, our results showed that this variable was not associated with any laceration group. The absence of documentation for the length of time with ruptured membranes is a weakness in our research which we acknowledge. Although literature data are unclear with respect to the involvement of this variable in the etiology of soft tissue laceration, Oliveira et al. concluded that a length of time with ruptured membranes of between 12 and 18 h before delivery plays a protective role in relation to mild perineal trauma [37].

Identification of risk factors that increase the odds of developing perineal injury is a matter of primary importance for young mothers. Measurements of pelvic dimensions and subpubic angle failed to predict obstetric anal sphincter injuries in certain studies [13,26]. At the same time, elastography research managed to prove that perineal laceration was more prevalent in women with stiffer perineal body [38]. Therefore, perineal preparation techniques for birth have been explored to avoid tears; instrument-assisted stretching and perineal massage managed to increase extensibility in pregnant women [39]. Future studies should focus on ways to assess the strength and distensibility of the perineal structures before and during labor to try to improve the accuracy in predicting lesions [14]. Reduced use of episiotomies was reported when practicing the hands-off technique in the second stage of labor, although no effects were noted on perineal tears [9].

Researchers in the United States estimated that pelvic floor disorders will be responsible for the increase in the health care costs associated with targeted treatment [15]. Future Romanian research should assess the clinical implications of enhancing the diagnostic accuracy of algorithms for obstetrical soft tissue trauma since this healthcare system already has a reduced potential of bearing additional financial burdens. The power of example can be seen with the publication of French guidelines for episiotomy, which led to an immediate decline in its practice although the correlated factors did not change; strategies for care improvement in obstetrics departments have managed to succeed in the proper implementation of these protocols [28].

We acknowledge the following limitations in our research: the absence of centralized and standardized birth registries in Romanian obstetrical practice, which supports possible data registration errors and hinders accurate estimates of perineal trauma based on their severity. In our research, specifically, features regarding labor induction or augmentation, labor length, use of fundal pressure, and epidural use were not considered, in part related to the first limitation and because of the increased bias potential. Maternal comorbidities known to compromise the perinatal outcome of adolescent mothers—obesity, anemia, hypertensive disorders, preterm labor, infections [8,24]—were not approached in this research for multiple reasons: the number of women associating certain complications was reduced and no statistical power could be obtained from analyzing the data; the heterogeneity of diagnostic conventions and protocols used in different institutions was identified as a potential source of bias in interpreting the results; and lastly, most pregnant women did not have comprehensive prenatal follow-up. The authors would like to concede the reduced number of early adolescents and are aware of this implication in the statistical interpretation of the results. All variables identified above are under consideration as subject to future research.

The strong points of this research reside first in the evaluation of birth-related lacerations in patients from two different geographical regions manifesting the same concerns on teenage vaginal birth comorbidities, and second in the in-depth observation of soft tissue lacerations and associated risk factors for specific adolescent age groups.

## 5. Conclusions

Among Romanian mothers who have spontaneous vaginal birth, the high rates of episiotomy are an indicative marker of continuous unselective use. This aspect is further concerning since adolescent mothers have a two times higher risk of developing vaginal and perineal tearing despite having episiotomy. The protective role of this incision could be verified only for superficial labial and periurethral lesions. Fortunately, the incidence of episiotomy tended to decrease with maternal age.

Primiparity was outlined as a risk factor for cervical tearing and for episiotomy practice in all women with spontaneous vaginal birth, irrespective of their age.

Adolescent mothers giving birth to neonates previously diagnosed with fetal macrosomia were not at risk for obstetrical soft tissue injury. Spontaneous rupture of membranes did not provide additional risk for intrapartum perineal tearing.

## Figures and Tables

**Table 1:** Demographic and obstetrical characteristics of women in the study groups.

Variable	Total No. of Patients (*n* = 1498)	Adolescent Mothers (Case Group) (*n* = 298)	Adult Mothers (Reference Group) (*n* = 1200)	*p*-Value
Age (years)	26.20 ± 6.86	17.30 ± 1.50	28.42 ± 5.81	Independent sample test: 0.00
Settlement: Rural Urban	899/1498 (60%) 599/1498 (40%)	194/298 (65.1%) 104/298 (34.9%)	705/1200 (58.8%) 495/1200 (41.3%)	Pearson Chi-square: >0.05 0.04
Gravidity 1 2–5 >5	406/1498 (27.1%) 844/1498(56.4%) 248/1498(16.5%)	182/298 (61.1%) 115/298 (38.5%) 1/298 (0.3%)	224/1200 (18.7%) 729/1200(60.8%) 247/1200(20.7%)	Likelihood Ratio: 0.00 >0.05 0.00
Parity 1 2 3 4 ≥5	482/1498 (32.2%) 404/1498 (27%) 252/1498 (16.8%) 142/1498 (9.5%) 218/1498 (14.7%)	203/298 (68.1%) 73/298 (24.5%) 17/298 (5.7%) 5/298 (1.7%) 0/298 (0.0%)	279/1200 (23.3%) 331/1200 (27.6%) 235/1200 (19.6%) 137/1200 (11.4%) 218/1200 (18.2%)	Likelihood Ratio: 0.00 >0.05 0.00 0.00 0.00
Pregnancy: single multiple	1497/1498(99.9%) 1/1498 (0.1%)	298/298 (100%) 0/298 (0.0%)	1199/1200(99.9%) 1/1200 (0.1%)	Fisher’s Exact Test: >0.05 >0.05
Presentation: cephalic breech	1493/1498(99.7%) 5/1498 (0.3%)	296/298 (99.3%) 2/298 (0.7%)	1197/1200(99.8%) 3/1200 (0.3%)	Fisher’s Exact Test: >0.05 >0.05
Membranes: intact ruptured	1220/1498(81.4%) 278/1498 (18.6%)	247/298 (82.9%) 51/298 (17.1%)	973/1200 (81.1%) 227/1200 (18.9%)	Pearson Chi-square: >0.05
History of diabetes	6/1498 (0.4%)	1/298 (0.3%)	5/1200 (0.4%)	Fisher’s Exact Test: >0.05
Fetal weight (g)	3115.1 ± 514.1	2976.5 ± 471.7	3149.5 ± 518.6	Independent Samples Test: 0.00
Apgar score: 8–10 1–7 0	1410/1498(94.1%) 80/1498 (5.3%) 8/1498 (0.5%)	274/298 (91.9%) 22/298 (7.4%) 2/298 (0.7%)	1136/1200(94.7%) 58/1200 (4.8%) 6/1200 (0.5%)	Pearson Chi-square: >0.05
Admission *: <5 days ≥5 days	1124/1197 (75.1%) 373/1197 (24.9%)	207/298 (69.5%) 91/298 (30.5%)	918/1200 (76.5%) 282/1200 (23.5%)	Pearson Chi-square: 0.012

* expressed in number of days of hospitalization.

**Table 2:** Distribution of group lacerations between the study participants according to their age groups.

Variable	Total No. of Patients (*n* = 1498)	Adult Mothers (Reference Group) (*n* = 1200)	Adolescent Mothers (Case Group) (*n* = 298)	*p*-Value (Case-Reference) Pearson Chi-Square	Early Adolescence (10–14 Years) (*n* = 11)	Middle Adolescence (15–17 Years) (*n* = 138)	Late Adolescence (18–19 Years) (*n* = 149)	*p*-Value (Adolescence Subgroups) Pearson Chi-Square
IST *	673 (44.9%)	598 (49.8%)	75 (25.2%)	0.00	1 (9.1%)	33 (23.9%)	41 (27.5%)	>0.05
Episiotomy	487 (32.5%)	320 (26.7%)	167(56.0%)	0.00	9 (81.8%)	80 (58.0%)	78 (52.3%)	>0.05
Laceration ** group I group II group III	46 (3.1%) 328 (21.9%) 109 (7.3%)	34 (2.8%) 254 (21.2%) 80 (6.7%)	12 (4.0%) 74 (24.8%) 29 (9.7%)	>0.05 >0.05 >0.05	0 (0.0%) 1 (9.1%) 1 (9.1%)	3 (2.2%) 34 (24.6%) 18 (13.0%)	9 (6.0%) 39 (26.2%) 10 (6.7%)	>0.05 >0.05 >0.05

* IST: intact soft tissue; ** lacerations: group I: injury of labia and periurethral region; group II: injury of vagina and perineum; group III: injury of cervix.

**Table 3:** Correlations between risk factors for obstetrical perineal tears and laceration groups.

Laceration Groups	Primiparity		Fetal Weight (≥4000 g)		History of Diabetes		Status of Membranes at Admission		
		*p* Value		*p* Value		*p* Value	Intact	Ruptured	*p* Value
**Group I lacerations:**									
Total	11 (23.9%)	0.22 *	0 (0.0%)	-	0 (0.0%)	-	34 (73.9%)	12 (26.1%)	0.18 *
Adolescent group	6 (50.0%)	0.20 **	0 (0.0%)	-	0 (0.0%)	-	8 (66.7%)	4 (33.3%)	0.13 *
Early adolescence	0 (0.0%)	-	0 (0.0%)	-	0 (0.0%)	-	0 (0.0%)	0 (0.0%)	-
Middle adolescence	3 (100.0%)	1.00 **	0 (0.0%)	-	0 (0.0%)	-	1 (33.3%)	2 (66.7%)	0.06 **
Late adolescence	3 (33.3%)	0.18 **	0 (0.0%)	-	0 (0.0%)	-	7 (77.8%)	2 (22.2%)	0.65 **
Adult group	5 (14.7%)	0.23 *	0 (0.0%)	-	0 (0.0%)	-	26 (76.5%)	8 (23.5%)	0.48 *
**Group II lacerations:**									
Total	118 (36%)	0.09 *	20 (6.1%)	0.01 *	2 (0.6%)	0.61 **	266(81.1%)	62 (18.9%)	0.85 *
Adolescent group	53 (71.6%)	0.45 *	3 (4.1%)	0.37 **	1 (1.4%)	0.24 **	63 (85.1%)	11 (14.9%)	0.55 *
Early adolescence	1 (100%)	1.00 **	0 (0.0%)	-	0 (0.0%)	-	1 (100.0%)	0 (0.0%)	1.00 **
Middle adolescence	24 (70.6%)	0.12 *	0 (0.0%)	-	0 (0.0%)	-	31 (91.2%)	3 (8.8%)	0.19 *
Late adolescence	28 (71.8%)	0.11 ***	3 (7.7%)	0.11 **	1 (2.6%)	0.26 **	31 (79.5%)	8 (20.5%)	0.55 *
Adult group	65 (25.6%)	0.31 *	17 (6.7%)	0.04 ***	1 (0.4%)	1.00 **	203(79.9%)	51 (20.1%)	0.59 *
**Group III lacerations:**									
Total	45 (41.3%)	0.03 *	1 (0.9%)	0.12 **	0 (0.0%)	-	91 (83.5%)	18 (16.5%)	0.57 *
Adolescent group	24 (82.8%)	0.07 *	0 (0.0%)	-	0 (0.0%)	-	23 (79.3%)	6 (20.7%)	0.60 **
Early adolescence	1 (100%)	1.00 **	0 (0.0%)	-	0 (0.0%)	-	1 (100.0%)	0 (0.0%)	1.00 **
Middle adolescence	15 (83.3%)	1.00 **	0 (0.0%)	-	0 (0.0%)	-	14 (77.8%)	4 (22.2%)	0.48 **
Late adolescence	8 (80.0%)	0.18 **	0 (0.0%)	-	0 (0.0%)	-	8 (80.0%)	2 (20.0%)	0.68 **
Adult group	21 (26.3%)	0.50 *	1 (1.3%)	0.24 **	0 (0.0%)	-	68 (85.0%)	12 (15.0%)	0.35 *

* Pearson Chi-square test; ** Fisher’s Exact Test; *** Pearson Chi-square with Bonferroni correction.

## Data Availability

The study database is available upon request.
